# Systemic induction of peroxidase and phenolic responses to UV radiation in *Nicotiana tabacum*

**DOI:** 10.1007/s00299-026-03985-5

**Published:** 2026-09-16

**Authors:** Zoltán Katona, Gyula Czégény, Kristóf Csepregi, Éva Hideg

**Affiliations:** Department of Plant Biology, University of Pécs, Ifjúság útja 6, Pécs, 7624 Hungary

**Keywords:** Ultraviolet (UV) radiation, Systemic response, *Nicotiana tabacum*, Class III peroxidase (POD) isozymes, Phenolic profiles, Antioxidant compounds

## Abstract

**Key message:**

Systemic changes in isoperoxidase profile and phenolic composition mirror those in leaves exposed to ultraviolet radiation, underscoring the essential role of systemic responses in plant health and development.

**Abstract:**

Plants respond to direct exposure to broadband ultraviolet (UV) light (280–400 nm) through localized protective and regulatory mechanisms. In our recent study using *Nicotiana tabacum* as a model plant, we showed that protective responses triggered by moderate, non-harmful UV doses could spread from directly treated leaves to UV-unexposed leaves situated above (systemic leaves). This study aimed to broaden the understanding of previous findings by investigating the similarities between direct and systemic plant responses, concentrating on two main aspects: class III peroxidase (POD) isozymes and the phenolic profiles of leaves. We compared the responses of directly UV-exposed and systemic leaves from the same plant with those of negative (completely UV-unexposed) and positive control (fully UV-exposed) plants. Native gel electrophoresis confirmed a general increase in POD activity in both direct and systemic UV responses, demonstrating similar rearrangements in POD isozyme patterns, which is a novel finding. Furthermore, high-performance liquid chromatography analyses of leaf extracts showed similar quantitative and qualitative alterations in phenolic profiles between UV-exposed leaves and systemic leaves. These changes included increased levels of potential POD substrates, such as chlorogenic acids and flavonoids, particularly quercetin-3-O-rutinoside, which serves as an excellent UV-absorbing compound and antioxidant. In general, our findings indicate that the extent and nature of the systemic UV response are similar to those triggered by direct exposure to UV radiation. These results highlight the potential of applying localized low-dose UV treatment to activate systemic antioxidant responses that affect plant health and their growth.

**Graphical abstract:**

**Figure:**
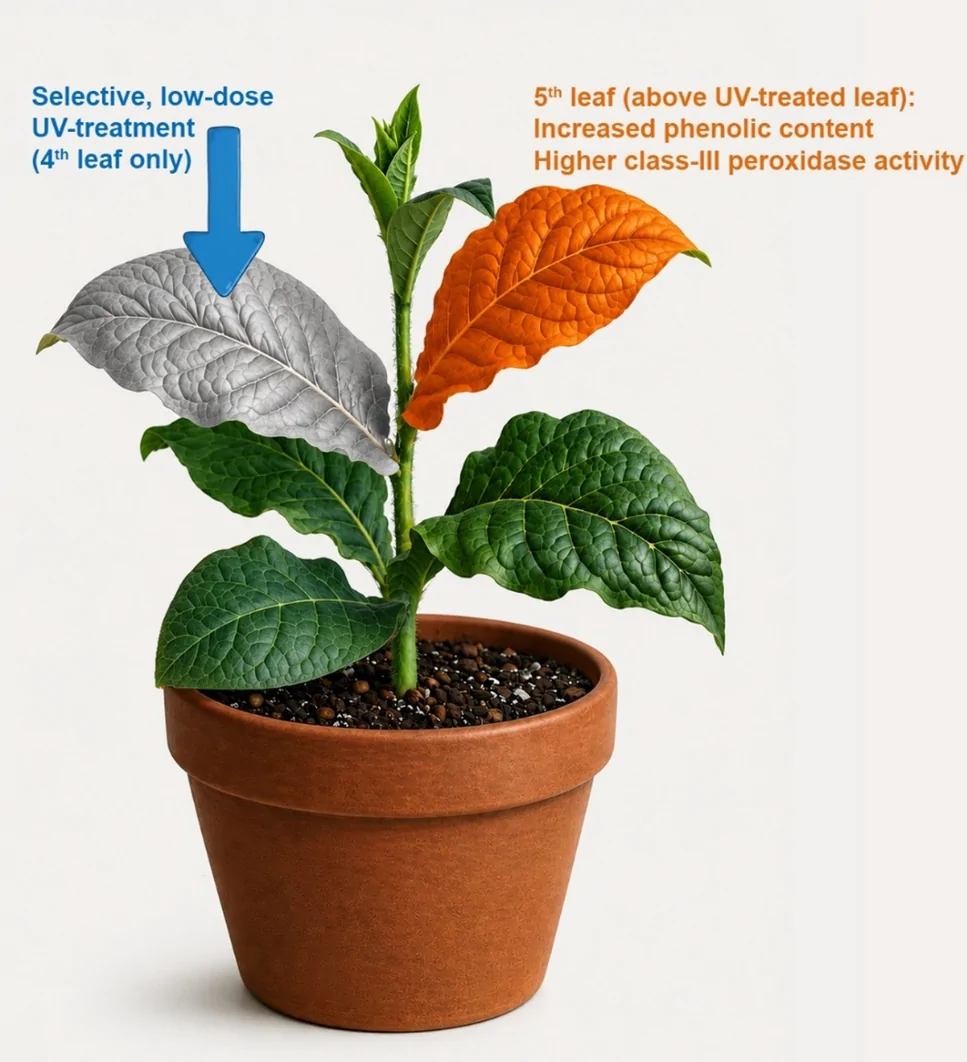

**Supplementary Information:**

The online version contains supplementary material available at https://doi.org/10.1007/s00299-026-03985-5.

## Introduction

Ultraviolet (UV) radiation, especially UV-B (280–315 nm), serves as a key environmental signal (Heijde and Ulm 2012; Jenkins 2014). UV-exposed plants respond with various localized protective and regulatory mechanisms, including upregulated flavonoid biosynthesis (Burchard et al. 2000), hormone modulation (Vanhaelewyn et al. 2016), and selective activation of antioxidant defenses (Czégény et al. 2016). Environmentally relevant UV-B levels cause eustress (Hideg et al. 2013), and UV pretreatment may increase abiotic stress tolerance (Jansen et al. 2019). Thus, targeted low-dose UV treatments offer the possibility of enhancing the levels of plant-protective and nutritionally beneficial secondary metabolites in indoor farming systems (Schreiner et al. 2014). UV-induced phenolic compound production and antioxidant stimulation are well documented; however, these responses have mostly been explored locally in the irradiated leaves. Recently, we showed that, similar to other abiotic factors, UV radiation has a systemic effect, manifesting as increased antioxidant capacity and photosynthetic activity in the leaf above the selectively UV-treated leaf of the same plant (Rácz et al. 2025). In general, systemic stress responses play a crucial role in plant growth by extending protective and regulatory mechanisms beyond the tissues directly affected by stressors. Systemic UV effects evoking beneficial metabolic effects similar to those of direct UV exposure would offer an excellent tool for enhancing the overall resilience and fitness of plants facing environmental challenges.

Using *Nicotiana tabacum* as a model organism, our prior investigation suggested the potential for the propagation of protective responses to non-irradiated regions, as evidenced by similar alterations in hydrogen peroxide levels, antioxidant enzyme activities, and epidermal UV absorption in both selectively UV-exposed 4th leaf and the 5th UV-unexposed leaf situated above it (Rácz et al. 2025). The present study broadens the scope of previous research by investigating the similarities in both direct and systemic plant responses, focusing on two key areas of UV response: peroxidase isozymes and phenolic profiles.

The first research question addressed the selective activation of class III peroxidase (POD) isozymes as a result of UV exposure (Rácz et al. 2018; Czégény and Rácz 2023). Here, we tested the hypothesis that both direct and systemic UV responses would produce comparable modifications in the POD isozyme pattern. We studied POD enzyme activities using native gel electrophoresis and compared them to the activities of leaves of plants wholly exposed to a higher dose of UV treatment supplementing growth PAR conditions. The second inquiry aimed to elaborate on and elucidate the previously documented systemic increase in epidermal ultraviolet (UV) absorbance. In our previous study (Rácz et al. 2025), we identified a systemic increase in leaf flavonoids using a non-invasive optical measurement technique (Goulas et al. 2004). This method, which evaluates the epidermal absorption of 365 nm photons, is considered a reliable estimate of the total leaf phenolic content (Siipola et al. 2015), although it is not suitable for mapping the variety of UV-absorbing phenolic acids and flavonoids present. In a recent study, we examined the quantitative and qualitative alterations in the phenolic composition of tobacco leaves induced by solar or artificial UV exposure (Csepregi et al. 2025). In the current investigation, we employed the same HPLC technique to compare the phenolic profiles of directly UV-exposed and systemic leaves of the same plant with those of negative control plants (entirely UV-unexposed) and positive control plants (entirely UV-exposed).

## Materials and methods

### Plant growth and treatments

Tobacco (*Nicotiana tabacum* L. cv. Petit Havana) plants were grown for 5 weeks post-emergence in a plant chamber (Sanyo MLR-352H-PE; Panasonic, Japan) equipped with a mixture of cold and warm light tubes (Polylux XL F36W/840 Cool White 4000 K and F36W/865 Daylight 6500 K, respectively; GE-Tungsram, Hungary). The growth conditions were set to a 16/8 h (day/night) photoperiod, 25/20 °C temperature regime, and a constant 65% humidity. Day conditions corresponded to approximately 110–125 μmol m^−2^ s^−1^ of photosynthetically active radiation (PAR). Ultraviolet (UV) treatments were conducted in two separate experiments using the same UV-B-centered broadband UV source (Q-Panel UVB-313EL, Q-Lab, Bolton, UK) covered with a single layer of cellulose diacetate filter (Courtaulds Chemicals, Derby, UK) to remove radiation below 280 nm.

In the first experiment, only the 4th true leaf of each plant was exposed to UV light for two consecutive days for 2 h daily (between 10 a.m. and noon) outside the growth chamber. This condition (referred to as UV1) corresponded to 6.8 kJ m^−2^ total biologically effective UV-B dose (Flint and Caldwell 2003). In this experiment, the 4th leaves were directly UV-exposed, and the systemic 5th leaves above these, and the 4th and 5th leaves of the control plants were used for comparison. Control plants were also removed from the growth chamber for 2 h, and their 4th leaves were placed under the same Q-panel tube. However, in these control experiments, the UV source was covered with an additional layer of UV-excluding filter foil (R3114; Roscolab, London, UK). Light conditions for systemic and control leaves have been previously reported, including spectral characterization (Rácz et al. 2025).

In the second experiment, whole plants were UV-exposed for 4 h daily (between 10 a.m. and 2 p.m.) for 4 consecutive days in a growth chamber equipped with the same Q-Panel UVB-313EL source. This treatment (referred to as UV2) provided a 27.4–30.2 kJ m^−2^ total biologically effective UV-B dose, supplementing the 110–125 μmol m^−2^ s^−1^ PAR. These irradiation conditions have been previously reported with spectral characterizations (Csepregi et al. 2025). Due to the alternate phyllotaxy of tobacco, the 5th leaves were 3–4 cm closer to the light sources than the 4th leaves; their exposure was slightly higher during the four treatment days. Control plants were kept in the same growth chamber but under a UV-excluding filter foil, as reported previously (Csepregi et al. 2025). Fourth and fifth leaves from UV-treated and control plants in the second experiment were used to create a reference for the first systemic experiment. Graphical outlines of the two experiments are shown in Supplementary Fig. S1.

### Native POD gel electrophoresis

Frozen leaves were ground into fine powder under liquid nitrogen. Each sample was mixed with an ice-cold buffer (50 mM sodium phosphate and 1 mM EDTA) and centrifuged at 21,380 × g for 45 min at 4 °C. Supernatants from four biological replicates per treatment group were pooled at equal volume ratios for electrophoresis. The protein content of the leaf extracts was determined according to Bradford (1976). Samples (20 µg protein per lane) were loaded onto a Novex™ 10%, Tris–Glycine Plus WedgeWell ™ Gel (Thermo Fisher Scientific, Waltham, MA, USA), and the PageRuler Plus Prestained Protein Ladder PN 26619 (Thermo Scientific, Waltham, MA, USA) was used as a molecular weight reference. Electrophoresis was performed for 2.5 h at 150 V and 50 mA using a Mini Gel Tank (Invitrogen by Thermo Fisher Scientific) and CS-300 V power supply (Cleaver Scientific Ltd, Rugby, Warwickshire, UK).

**Fig. 1:**
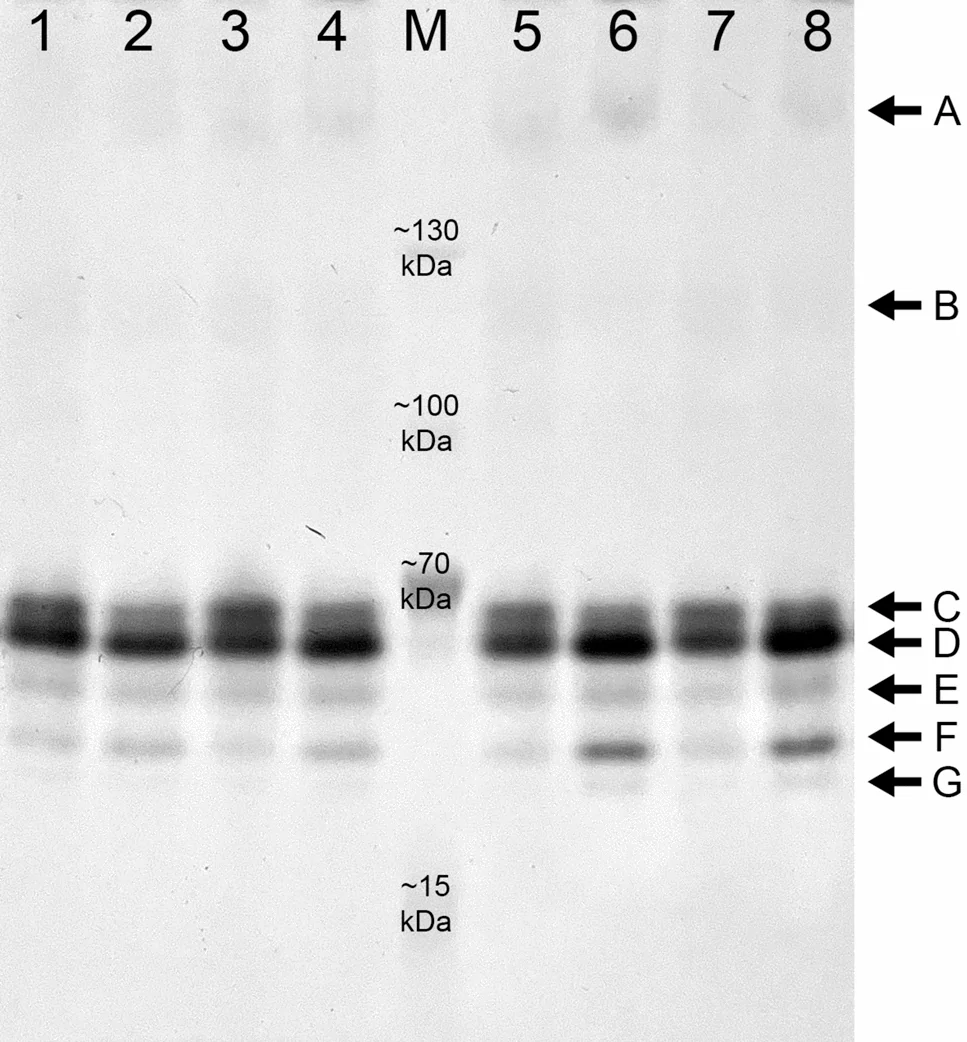
Native PAGE zymogram of class III peroxidase isoenzymes in tobacco (*Nicotiana tabacum* L. cv. Petit Havana) leaves following local and systemic UV exposure. Leaf extracts from two independent experiments (lanes 1–4, ‘UV1’; lanes 5–8, ‘UV2’) were separated on a non-denaturing polyacrylamide gel. The lanes are numbered as follows: 1, control 4th leaf; 2, UV1 4th leaf; 3, control 5th leaf; 4, systemic 5th leaf; M, protein ladder; 5, control 4th leaf; 6, UV2 4th leaf; 7, control 5th leaf; 8, UV2 5th leaf

After electrophoresis, POD activity staining was performed at room temperature, as described previously (Rácz et al. 2018). The gel was first rinsed with distilled water and then incubated in 10 mM H_2_O_2_. This was followed by incubation in 1 mg/mL 2,2’-azino-bis (3-ethylbenzthiazoline−6-sulfonic acid) (ABTS) in phosphate-citrate buffer (pH 5.0) until blue–green bands of oxidized ABTS became visible in ca. 5–10 min. The digital photograph was converted to grayscale image and quantified using Fiji open-source software (Schindelin et al. 2012). Two high-activity bands (~ 65 kDa in lanes 6 and 8, Fig. 1) became slightly oversaturated, exceeding the detection threshold of the software, and the resulting inaccurate histogram peaks were replaced with values derived from Gaussian curve fitting in OriginPro (Version 2026, OriginLab Corporation, Northampton, MA, USA). As the activity ratios were not directly quantified, this substitution did not impact the conclusions. The unprocessed digital photograph is shown in Supplementary Fig. S2.

### Analysis of phenolic compounds with liquid chromatography (HPLC–DAD)

Leaf samples were rapidly frozen in liquid nitrogen and stored at − 60 °C until further processing. The frozen material was subsequently lyophilized and extracted using aqueous methanol (MeOH:H_2_O, 70:30, v/v). The extracts were sonicated for 15 min in an ultrasonic bath (RoHS JP-020, Shenzhen, China), followed by centrifugation at 15,000 × g (Thermo Fisher Scientific Inc., Waltham, MA, USA) for 10 min at room temperature. The supernatants were collected, and the remaining pellets were re-extracted with 350 µL of the same solvent for each tube. This extraction step was repeated twice, and the supernatants from all three extraction cycles were combined for each sample (corresponding to one leaf per plant). An aliquot of 1 mL from each pooled extract was filtered through a 0.2 µm PTFE membrane filter (Lab-Ex, Hungary) and stored at −20 °C until HPLC analysis. Chromatographic separation of phenolic metabolites was performed using a Shimadzu LC-20 high-performance liquid chromatography (HPLC) system (Shimadzu, Kyoto, Japan) equipped with a diode array detector (DAD). Analyses were performed using a Kinetex Core–Shell C18 column (100 mm × 4.6 mm, 2.6 µm particle size, Phenomenex, Germany). The mobile phase consisted of solvent A (0.5% formic acid in distilled water) and solvent B (0.5% formic acid in methanol). Elution was achieved using the following gradient program: 0–2 min 0% B, 2–6 min 10% B, 6–30 min 30% B, 30–35 min 80% B, 35–37 min 100% B, 37–39 min 100% B, 39–43 min 0% B, 43–45 min 0% B for column equilibration. The flow rate was maintained at 0.9 mL min^−1^, and the injection volume was set to 7 μL. Phenolic compounds were detected using diode array detection at appropriate wavelengths depending on the compound class (phenolic acids: 320 nm, flavonoids: 345 nm). Reference compounds were purchased from Extrasynthese (Lyon, France).

### Statistical analyses

**Fig. 2:**
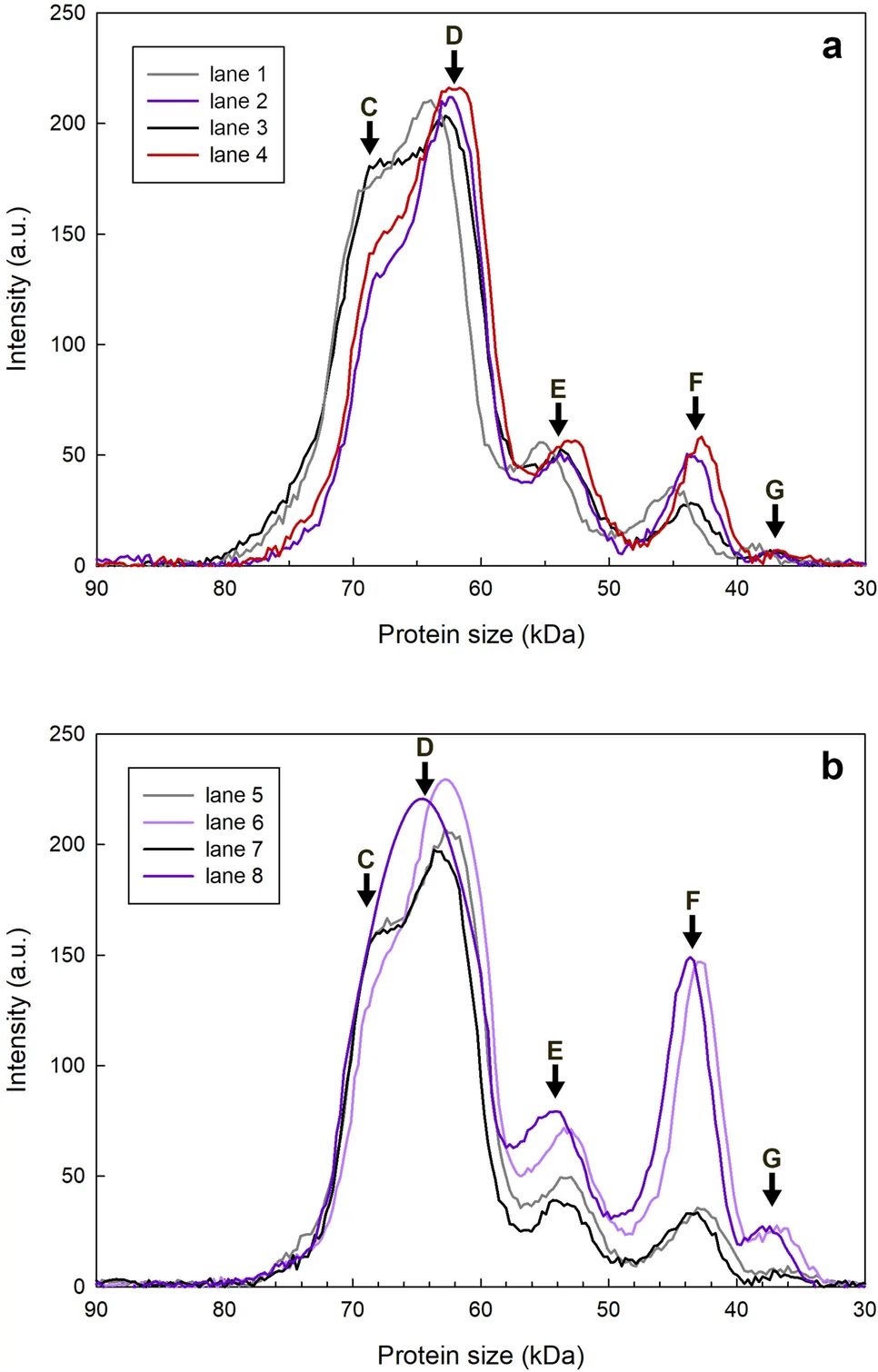
Densitograms illustrating changes in class III peroxidase isoenzyme activities measured in the leaves of either (**a**) partially or (**b**) totally UV-exposed tobacco plants

**Fig. 3:**
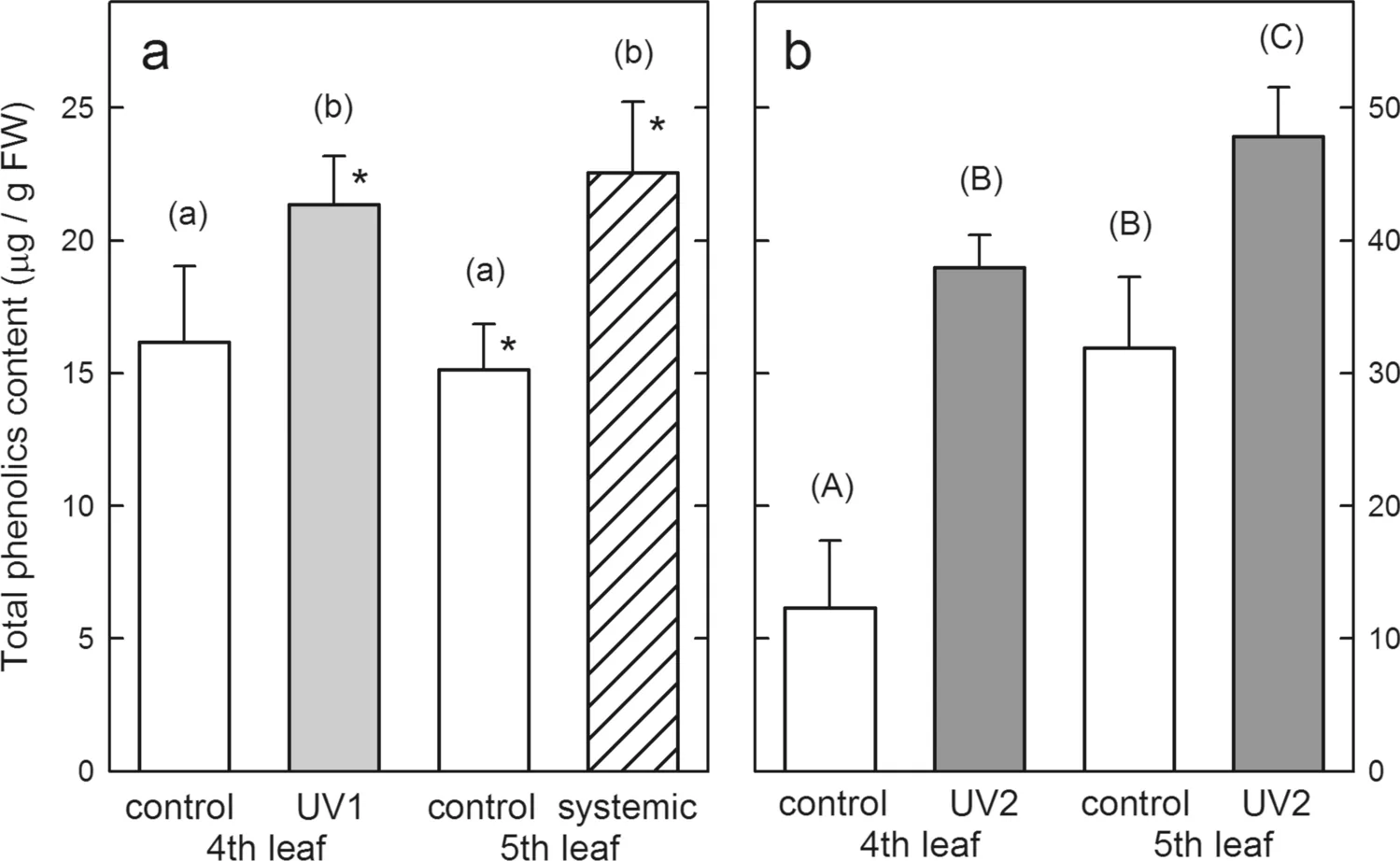
Total phenolic content of leaves of (**a**) partially and (**b**) totally UV-exposed tobacco plants. Column heights and error bars indicate the means and standard deviations (*n*=4–6). The white columns represent the UV-unexposed control leaves in both experiments. Light and dark gray columns correspond to the UV1 treatment applied to the 4th leaf only and to the UV2 treatment applied to the whole plant, respectively. Dashed columns indicate systemic 5th leaves above the directly UV-exposed 4th leaves. See Materials and methods for treatment details. Lower- and upper-case letters in parentheses show the results of the statistical analysis of the lower- and higher-UV-dose experiments, respectively; different letters indicate significantly (*p* < 0.05) different means. Asterisks in the left panel indicate significantly (*p* < 0.05) different means when the corresponding samples were compared in the two experiments

**Fig. 4:**
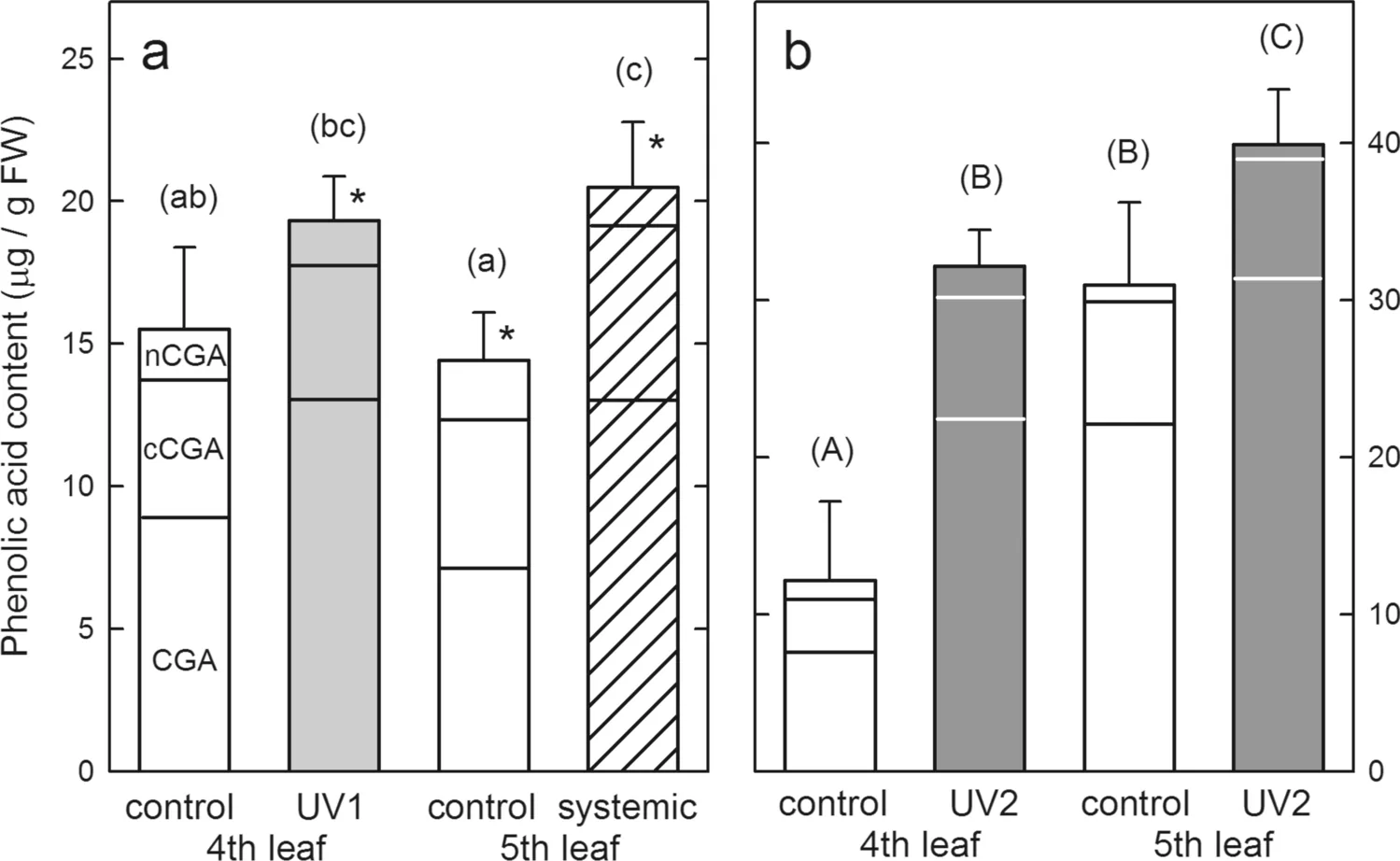
Leaf phenolic acid content of either (**a**) partially or (**b**) totally UV-exposed tobacco plants. The component distributions are indicated by vertical divisions in each column. CGA, chlorogenic acid; cCGA, crypto-chlorogenic acid; nCGA, neo-chlorogenic acid. See the legend of Fig. 3 for further details

**Fig. 5:**
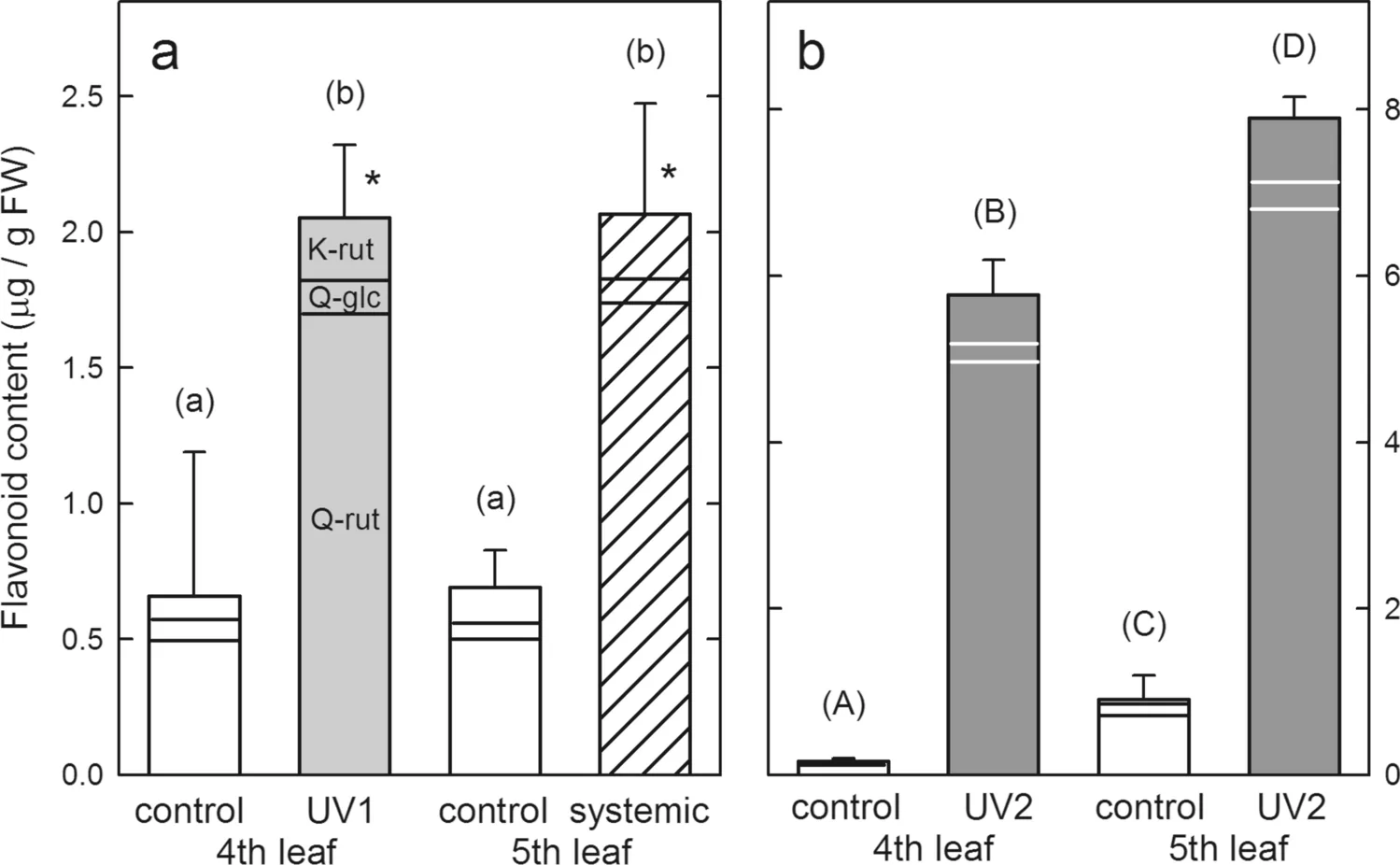
Leaf flavonoid contents of either (**a**) partially or (**b**) totally UV-exposed tobacco plants. The component distributions are indicated by vertical divisions in each column. K-rut, kaempferol-3-*O*-rutinoside; Q-glc, quercetin-3-*O*-glucoside; Q-rut, quercetin-3-*O*-rutinoside. See the legend of Fig. 3 for further details

Phenolic profile data were analyzed using the PAST software (Hammer et al. 2001). The data from the two UV experiments were analyzed separately and comparatively. Separate analysis included four data groups and used one-way ANOVA to examine the null hypothesis that all group means were equal. When the null hypothesis was rejected at *p* < 0.05, Tukey’s post hoc test was used to compare the four means, and different means were assigned different letters in the corresponding figures. These indicative letters in parentheses are lower case for data from the systemic experiment using the UV1 dose and upper case for the whole-plant exposure experiment using the UV2 dose. Comparative analysis used two-sample *t *tests to examine the null hypothesis that the two group means (i.e., data of UV1 and UV2 exposed 4th leaves) were the same. In Figs. 2–5, an asterisk above the column representing UV1 data indicates whether the null hypothesis was rejected at *p* < 0.05 level.

## Results

Native gel electrophoresis identified seven POD isoforms with distinct apparent molecular weights. Bands corresponding to the various isoforms are labeled A–G and marked with arrows in Fig. 1. The isoforms with the strongest affinity for ABTS as a substrate were in the 40–75 kDa range (bands C–F); however, two isoforms with higher (A and B, approximately 160 and 125 kDa, respectively) and one isoform with lower molecular weight (G, approximately 35 kDa) showed weaker activity. Owing to their weak representation, isoforms A and B were not compared among the samples. Figure 1 compares POD activities in two distinct experiments: short, targeted exposure of the 4th leaf only to the lower dose UV (UV1, left side) and longer whole-plant acclimation to a higher dose of UV (UV2, right side). UV-unexposed control plants had similar POD patterns in the 4th (lanes 1 and 5) and 5th (lanes 3 and 7) leaves, displaying the strongest activities in isoforms C and D. The 4-day acclimation of whole plants to the UV2 dose had similar effects on both studied leaves, leading to enhanced activities of isoforms D–G and a slight decrease in band C. The effects of the two distinct UV exposures were compared to address the following questions: (i) Does the four-times lower UV1 dose evoke similar changes in the POD isoform pattern of the 4th leaves as the prolonged acclimation to UV2, and (ii) can the exclusive exposure of the 4th leaf to the dose UV1 evoke a systemic POD response in the UV-unexposed 5th leaf. Figures 1 and 2 demonstrate that the responses to both questions were affirmative. The most characteristic response to direct UV exposure was the increase in POD activities in bands D and G. A comparison of lanes 2 and 6 shows that the 4th leaves responded to both UV1 and UV2 with these features. The enhancement of lower-molecular-weight bands E and G, accompanied by a decrease in band C, was only observed in response to UV2 and was either missing or less pronounced in UV1 exposed leaves, suggesting that these require a higher UV flux or more prolonged exposure. Also, a comparison of lanes 2 and 4 confirmed the previously reported systemic UV effect (Rácz et al. 2025) and demonstrated that the isozyme pattern of the systemic POD response of the 5th leaf was similar to the direct UV-evoked response of the 4th leaves.

The total content of extractable leaf phenolics is shown in Fig. 3. HPLC analysis showed that even low-dose UV treatment (UV1) increased the total phenolic content in the 4th leaf and confirmed a systemic increase in the 5th leaf. Moreover, the relative extent of increase was similar, approximately 35–50% in both leaves under UV1 conditions (Fig. 3a). The magnitude of the increase was consistently higher when whole plants were acclimated to the four-times higher dose (UV2); nevertheless, similar trends were observed in both experimental setups. Detailed HPLC analysis identified chlorogenic acids (5-*O*-caffeoylquinic acid, CGA) as the main phenolic compound in all the leaves. Depending on the treatment conditions, CGA, crypto-chlorogenic acid (4-*O*-caffeoylquinic acid, cCGA), and neo-chlorogenic acid (3-*O*-caffeoylquinic acid, nCGA) gave 80–97% of the total phenolic content (Fig. 4). The relative proportions of these compounds showed only minor variations between the treatments, whereas their concentrations followed the trends observed for the total phenolic content. The UV-induced increase was mainly observed in CGA, whereas cCGA and nCGA showed smaller changes, particularly under the UV1 conditions. In both experiments, higher values were observed under UV2, with CGA showing the most pronounced increase. The contributions of individual CGA isomers remained similar across treatments, with CGA being the dominant phenolic acid in all the samples.

Although flavonoids represented only a small proportion (3–5%) of the total phenolic content in control leaves, these compounds were more UV-inducible than phenolic acids. Tobacco leaf flavonoids are primarily flavonols, with quercetin-3-*O*-rutinoside (Q-rut) as the dominant compound. Quercetin-3-*O*-glucoside (Q-glc) and kaempferol-3-*O*-rutinoside (K-rut) were also identified as minor components. The increase, both UV-inducible and systemic, was detectable in all identified flavonols but was the strongest in Q-rut content (Fig. 5). All control leaves had similar, although not identical, total and main flavonoid contents. Unlike in the first experiment (controls to UV1), where there were no significant differences, in the second experiment (controls to UV2), the 5th leaves had higher phenolic levels than the 4th leaves. The total flavonoid content tripled in response to the low-dose UV1 treatment in the 4th leaves and increased similarly in the systemic 5th leaves (Fig. 5a). The four-times higher UV2 dose caused a much larger, approximately 30-fold increase in the 4th leaves (Fig. 5b).

## Discussion

Flavonoids are one of the largest classes of plant secondary metabolites and are involved in several physiological functions, including UV protection (Winkel-Shirley 2001). Together with phenolic acids, these compounds protect against oxidative stress in various ways. Both chlorogenic acid (CGA) and quercetin derivatives support class III peroxidase (POD) enzymes as substrates (Rácz et al. 2020; Czégény and Rácz 2023) and have high ROS reactivities (Csepregi and Hideg 2018). In this study, we showed that the levels of these phenolics and the activities of several POD isoforms can be significantly increased not only by direct UV treatment but also systematically. Sample separation on native gel revealed UV-triggered alterations in the peroxidase profile of tobacco in both directly illuminated and systemic leaves (Figs. 1 and 2). Class III peroxidases are a large multigene superfamily, and monomeric enzyme proteins typically range from 28 to 60 kDa in general (Freitas et al. 2024) and 30–50 kDa in tobacco (Lagrimini and Rothstein 1987; Klotz et al. 1998; Passardi et al. 2005; Kukavica et al. 2012). However, high glycosylation or protein aggregation can increase the apparent molecular mass, resulting in slower migration (Veitch 2004). Accordingly, we identified two relatively low-activity bands (A and B in Fig. 1), which likely represent highly glycosylated POD isoenzymes. The main cluster of tobacco PODs was present in the lower, 70–40 kDa range (bands C–F). Both lower UV1 and higher UV2 doses increased the activities of bands D–F and decreased the activity of band C (70 kDa) (Figs. 1 and 2). This reorganization may occur for multiple reasons: (i) bands C and D–F represent the products of distinct POD-coding genes, or (ii) UV radiation alters the post-translational modifications of peroxidases, inducing alternative glycosylation and migration of the same gene product. Irrespective of the underlying cause for this selective enhancement and rearrangement of the POD isozyme pattern, similar mechanisms were observed in systemic 5th leaves (lane 4, Fig. 2a). This observation suggests that the signals responsible for these modifications were disseminated throughout the plant, thereby inducing UV-naïve leaves to initiate a preemptive defense response.

Enhancing the activities of these defensive enzymes alone would not be sufficient, as these enzymes require adequate substrate availability to function effectively. The whole-plant irradiation experiment demonstrated that a relatively low UV dose (27–30 kJ m^−2^ biologically effective UV-B, administered for 4-d, 4 h daily) led to a significant increase in phenolic compounds (Fig. 3b), many of which serve as potential substrates for POD enzymes. In this experiment, the relative UV-inducible increase in CGA and thus in total phenolic content was lower in the 5th leaf than in the 4th leaf, which may be explained by differences in base levels being higher in the 5th leaf than in the 4th leaf in the absence of UV. (Figs. 3b, 4b). Such a difference was not observed between the control leaves of the lower dose (UV1) experiment when the plants were removed from the growth chamber for the duration of the treatment, in contrast to the higher dose experiment (UV2), in which plants were kept in the chamber all the time. Consequently, although neither control group received UV light, their PAR conditions were different during the 2×2 h of the experiments. The cause of this phenomenon and its impact on phenolic base levels remain unknown. However, as this variation did not influence our conclusions regarding systemic or direct UV effects, the underlying reasons were not investigated in this study.

In a recent study, using the same irradiation conditions and tobacco leaves of the same age, we showed that 4-d of acclimation to this UV dose did not decrease photosynthesis (Csepregi et al. 2025). The present experiments further illustrate that even a reduced, quarter dose of UV radiation can significantly enhance the phenolic content of leaves (Fig. 3a). Moreover, the accumulation of these secondary metabolites was not restricted to the 4th leaf directly exposed to UV radiation but was also observed in the 5th leaf, indicating a whole-plant response to localized UV treatment. HPLC analysis confirmed that UV radiation increased both phenolic acid and flavonoid content, both locally and systemically, and altered their relative proportions. Although chlorogenic acids remained the dominant phenolic components, flavonoids showed the greatest relative increase in content. Direct UV responses in phenolic compounds were dose-dependent and consistent with previous observations that UV radiation preferentially induces flavonoid biosynthesis and modifies flavonol composition in favor of quercetin derivatives in several other species (Olsson et al. 1998; Ryan et al. 1998; Götz et al. 2010; Jenkins 2014; Schreiner et al. 2014). Systemic changes followed similar patterns, and although these were very similar to the direct UV responses, it cannot be concluded that UV exposure of the 4th leaf would specifically prime the 5th leaves to defend against UV stress, similar to sunfleck high PAR exposures prompting defense against photoinhibition (Karpinski et al. 1999; Jiang et al. 2020). Increased production of chlorogenic acid and flavonoids has been documented in tobacco leaves under other stress conditions (Torras-Claveria et al. 2012; Wang et al. 2023a; Yin et al. 2025), and key enzymes of phenylpropanoid biosynthesis have been reported to be upregulated by several environmental factors, such as high PAR and low temperatures (Voght 2010; Ortiz et al. 2023). The shift in flavonol ratios in favor of quercetin derivatives is known to be due to the selective activation of the flavonoid 3'-hydroxylase (F3′H) enzyme in UV-exposed leaves (Ryan et al. 2002; Schoenbohm et al. 2002). However, this enzyme, which also acts on several flavonoids other than kaempferols, has been shown to be upregulated by other stress conditions as well (Walia et al. 2005; Lillo et al. 2008; Olsen et al. 2009), because dihydroxylated quercetins are better antioxidants than monohydroxylated kaempferols (Rice-Evans et al. 1996; Csepregi and Hideg 2018). Thus, the altered phenolic profile of systemic leaves may represent a general preemptive stress response, rather than a specific preparation for subsequent UV exposure.

The increase in phenolic compounds in the systemic leaves can be explained by either increased local production or the transport of these compounds. Flavonoids are synthesized on the cytosolic face of the ER membrane and are then subjected to various modifications such as glycosylation (Zhao and Dixon 2010). Flavonoid transport has been explored in detail in the case of colored molecules, such as anthocyanins, and there is evidence for vesicle-mediated and specific Multidrug And Toxic Compound Extrusion (MATE)-type protein-mediated transport of these flavonoids into the vacuoles. (Zhao and Dixon 2010). To the best of our knowledge, there is no evidence for the similar transport of phenolic acids or colorless flavonols, such as quercetins. Therefore, local upregulation of phenolic biosynthesis in systemic leaves triggered by a mobile signal originating from the directly UV-exposed leaf is a more probable mechanism.

Although it is plausible that the increases in POD isoform activity and phenolic metabolite content are co-regulated, their simultaneous increase does not confirm this relationship. To date, the components of systemic UV signaling remain largely unknown. We previously showed the involvement of hydrogen peroxide (Rácz et al. 2025); however, the participation of other common signaling molecules, such as plant hormones, nitric oxide, or calcium, is also possible. Direct H_2_O_2_ treatment enhances the biosynthesis of phenolic compounds by stimulating multiple genes involved in phenylpropanoid biosynthesis in lettuce leaves (Wang et al. 2023b). There are also indications that POD proteins can also be directly differentially regulated by H_2_O_2_ in rice roots (Zhou et al. 2011). To further this research, it is essential to explore the source of H_2_O_2_ appearing as part of the systemic UV signal, whether it is organellum-derived (such as chloroplasts or peroxisomes). Second, it is important to study the involvement of signaling components beyond H_2_O_2_, which are recognized for their contributions to other H_2_O_2_-related signaling pathways in plants (Petrov and Van Breusegem 2012). Finally, by focusing on the interconnectivity of H_2_O_2_, POD, and phenolic metabolites, we should investigate the potential roles of flavonol catabolites (Bozzo and Unterlander 2021) in the systemic UV signaling process.

## Conclusion

Plants do not function as a collection of autonomous leaves but as an integrated network hub capable of well-organized systemic coordination. Systemic stress responses play a crucial role in plant growth by extending protective and regulatory mechanisms beyond tissues directly impacted by stressors such as UV radiation. This coordinated signaling enables non-exposed parts of the plant to proactively activate defenses, such as antioxidant enzyme and phenolic compound production, which help mitigate oxidative damage and maintain physiological balance. By facilitating communication between leaves, systemic responses ensure a comprehensive adaptation strategy that enhances the overall resilience and fitness of plants in response to environmental stress. This broader protective scope supports survival and growth by preparing the entire organism to withstand fluctuating stress conditions rather than relying solely on localized reactions. We illustrated this using *Nicotiana tabacum*, where systemic changes in POD enzyme and phenolic profiles mirrored those in directly UV-exposed leaves, underscoring the significance of systemic responses in maintaining plant health and growth.

## Supplementary Information

Below is the link to the electronic supplementary material.

Supplementary file1 (PDF 316 KB)

## Data Availability

The datasets generated and/or analyzed during the current study are available from the corresponding author upon reasonable request.
