# Supplementary material for: Systemic induction of peroxidase and phenolic responses to UV radiation in Nicotiana tabacum
Source: Plant Cell Rep. 2026 Sep 16;45(10):292. doi: 10.1007/s00299-026-03985-5 (PMC13582051; doi:10.1007/s00299-026-03985-5)
Supplement: Supplementary file 1 [file 299_2026_3985_MOESM1_ESM.pdf]

Supplementary Material

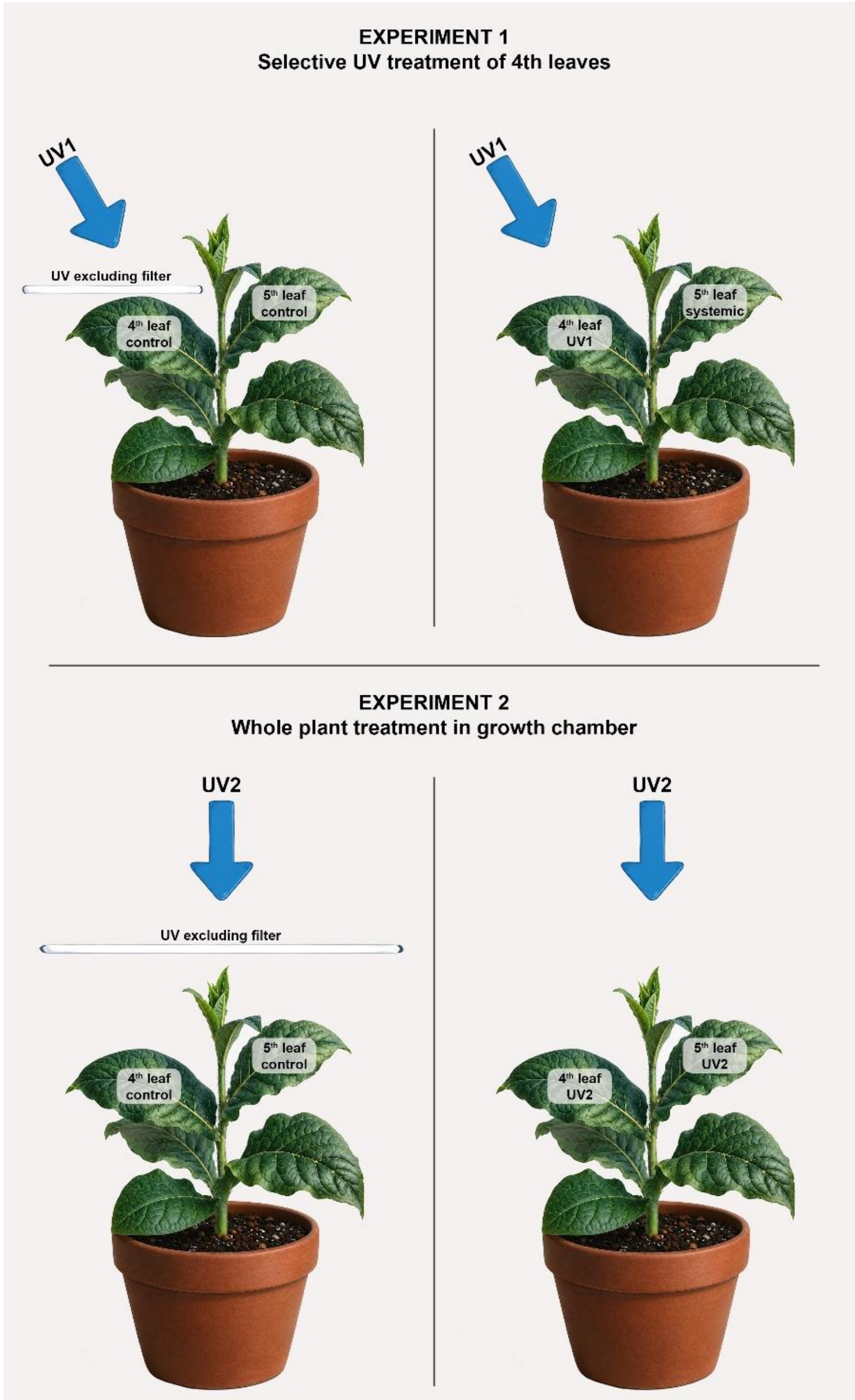

Supplementary Figure S1

Graphical representation of the two experiments that served as the basis of this study. UV1, 6.8 kJ m^{-2} total biologically effective UV-B; UV2, 27.4-30.2 kJ m^{-2} total biologically effective UV-B. Please refer to the Materials and Methods section for further details.

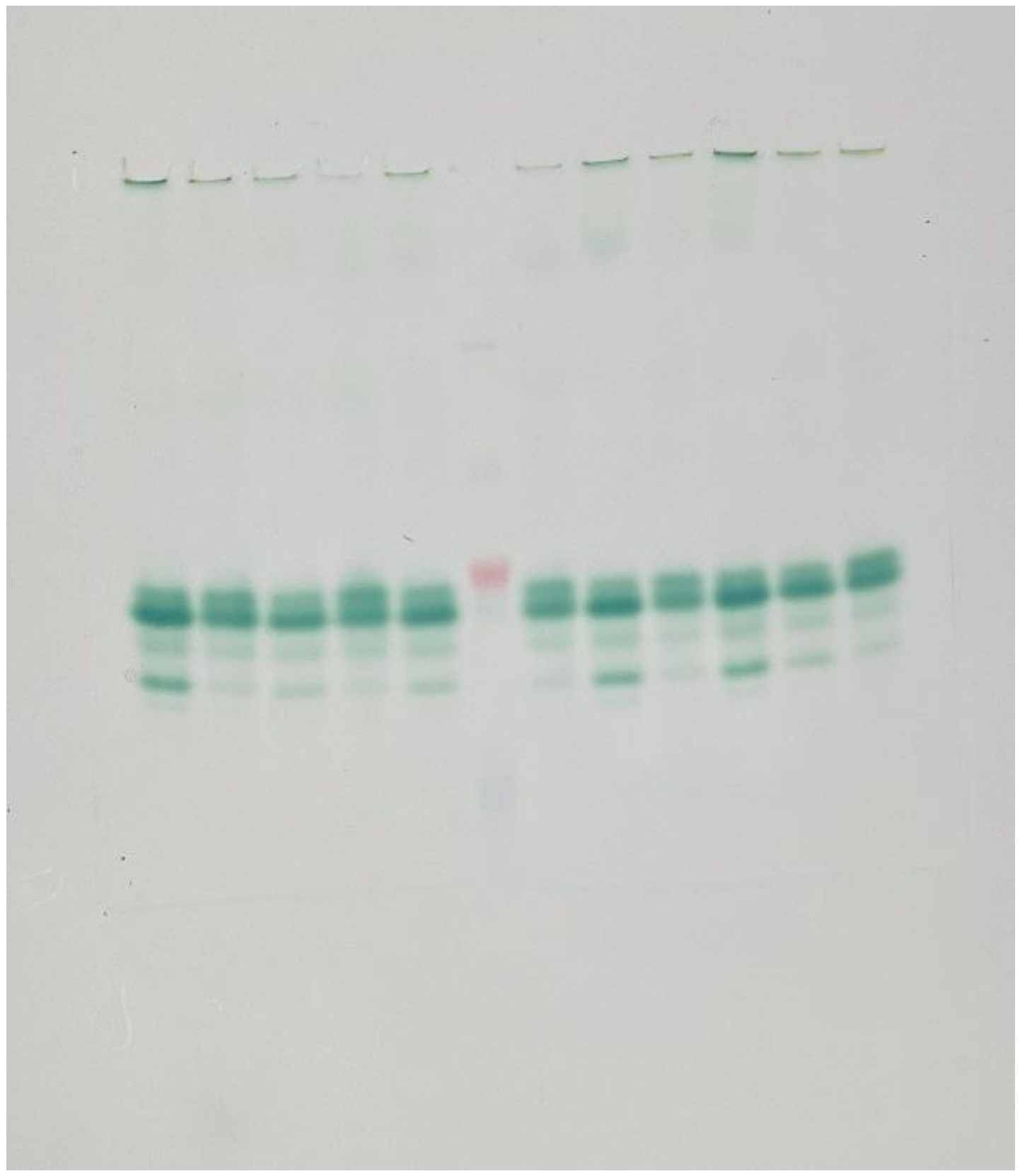

Supplementary Figure S2
Original native PAGE zymogram showing peroxidase (POD) activity in tobacco (*Nicotiana tabacum*) leaves under varying UV treatment regimes.
The gel was stained for POD activity using ABTS as a substrate. Three lanes at the edges (first, eleventh, and twelfth), which were repetitions, were not used in the analyses presented in Fig. 2.
Lanes were loaded as follows (from left to right):

- UV2 experiment: UV-exposed 5th leaf (not used)
- UV1 experiment: control 4th leaf (labelled as lane 1 in Figs. 1 and 2)
- UV1 experiment: UV-exposed 4th leaf (lane 2 in Figs. 1 and 2)
- UV1 experiment: control 5th leaf (lane 3 in Figs. 1 and 2)
- UV1 experiment: systemic 5th leaf (lane 4 in Figs. 1 and 2)
- protein ladder
- UV2 experiment: control 4th leaf (lane 5 in Figs. 1 and 2)
- UV2 experiment: UV-exposed 4th leaf (lane 6 in Figs. 1 and 2)
- UV2 experiment: control 5th leaf (lane 7 in Figs. 1 and 2)
- UV2 experiment: UV-exposed 5th leaf (lane 8 in Figs. 1 and 2)
- UV1 experiment: UV-exposed 4th leaf (not used)
- UV2 experiment: control 5th leaf (not used)

Please refer to the Materials and Methods section for further details.
